# F Plasmid Lineages in Escherichia coli ST95: Implications for Host Range, Antibiotic Resistance, and Zoonoses

**DOI:** 10.1128/msystems.01212-21

**Published:** 2022-01-25

**Authors:** Max Laurence Cummins, Cameron J. Reid, Steven Philip Djordjevic

**Affiliations:** a The iThree Institute, University of Technology Sydney, Ultimo, NSW, Australia; b Australian Centre for Genomic Epidemiological Microbiology, University of Technology Sydney, Ultimo, NSW, Australia; Istanbul Medipol University School of Medicine

**Keywords:** ST131, ST73, ST95, *E. coli*, APEC, antibiotic resistance, antimicrobial resistance, ColV, *Escherichia coli*, ExPEC, plasmid, pUTI89

## Abstract

Escherichia coli sequence type 95 (ST95) is an extraintestinal pathogenic E. coli (ExPEC) renowned for its ability to cause significant morbidity and mortality in humans and poultry. A core genome analysis of 668 ST95 isolates generated 10 clades (A to J), 5 of which are reported here for the first time. F plasmid replicon sequence typing showed that almost a third (178/668 [27%]) of the collection carry pUTI89 (F29:B10) and were restricted to clade A and a sublineage of clade B. In contrast, almost half (328/668 [49%]) of the collection across multiple clades harbor ColV plasmids (multiple F types). Strikingly, ST95 lineages with pUTI89 were almost exclusively from humans, while ColV^+^ ST95 lineages were sourced from poultry and humans. Clade I was notable because it comprises temporally and geographically matched ColV^+^ isolates sourced from human and retail poultry meat, suggesting interspecies transmission via food. Clade F contained ST95 isolates of bovine origin, none of which carried ColV or pUTI89 plasmids. Remarkably, an analysis of a cohort of 34,176 E. coli isolates comprising 2,570 sequence types mirrored what was observed in ST95: (i) pUTI89 was overwhelmingly linked to E. coli sourced from humans but almost entirely absent from 13,027 E. coli isolates recovered from poultry, pigs, and cattle, and (ii) E. coli isolates harboring ColV plasmids were from multiple sources, including humans, poultry, and swine. Overall, our data suggest that F plasmids influence E. coli host range, clade structure, and zoonotic potential in ST95 and ExPEC more broadly.

**IMPORTANCE** E. coli ST95 is one of five dominant ExPEC lineages globally and noted for causing urinary tract and bloodstream infections and neonatal meningitis in humans and colibacillosis in poultry. Using high-resolution phylogenomics, we show that F replicon sequence type is linked to ST95 clade structure and zoonotic potential. Specifically, human centric ST95 clades overwhelmingly harbor F29:B10 (pUTI89) plasmids, while clades carrying both human- and poultry-sourced isolates are typically ColV^+^ with multiple replicon types. Importantly, several clades identified clonal ColV^+^ ST95 isolates from human and poultry sources, but clade I, which housed temporally and spatially matched isolates, provided the most robust evidence. Notably, patterns of association of F replicon types with E. coli host were mirrored within a diverse collection of 34,176 E. coli genomes. Our studies indicate that the role of food animals as a source of human ExPEC disease is complex and warrants further investigation.

## INTRODUCTION

Escherichia coli is one of the most frequently isolated Gram-negative pathogens globally. Broadly speaking, members of the species can be divided into two primary pathotypes named according to the anatomical sites they infect: intestinal pathogenic E. coli (IPEC) and extraintestinal pathogenic E. coli (ExPEC). ExPEC colonizes the mammalian gut and typically does not cause gastrointestinal disease but can cause disease at extraintestinal sites when afforded the opportunity. In humans, ExPEC causes urinary tract infections (UTIs), blood infections (bacteremia and sepsis), soft tissue infections, neonatal meningitis (1–4), and ventilator-associated pneumonia (5). These pathologies are responsible for billions of dollars in health care expenditure each year and cause significant morbidity and mortality (6).

Despite this, the understanding of how ExPEC emerge, adapt, and spread is poor. Many sequence types (STs) of E. coli are known to cause extraintestinal infection; however, relatively few including ST131, ST69, ST95, ST73, ST10, ST405, and ST38, are responsible for the majority of the ExPEC disease burden (7). Much attention has been given to ExPEC lineages that circulate within hospital environments, such as the global pandemic extended-spectrum β-lactamase (ESBL)-producing and fluoroquinolone-resistant (FQR) ST131 *fimH30*-Rx lineage. However, UTIs are predominantly community-onset diseases, suggesting that reservoirs of ExPEC that adversely impact human health reside outside health care settings. Apart from the human gastrointestinal tract, companion and food animals, sewage, and retail meats have all been implicated (8). E. coli isolates that are genetically closely related to isolates from human ExPEC infections have also been recovered from synanthropic birds, wild animals, and natural environments (9–13). In commercial poultry production, a subset of ExPEC known as avian-pathogenic E. coli (APEC) cause colibacillosis, a disease that imposes a major economic cost to the poultry industry. Several notable APEC sequence types, such as ST95 and ST117, also cause human extraintestinal diseases (14, 15). Furthermore, foodborne extraintestinal diseases affecting humans have been touted for many years (16). For example, unlike the pandemic clade C lineages of ST131 that carry an H30 *fimH* allele, ST131 H22 (clade B) are known to harbor ColV F virulence plasmids (11, 17). A high-resolution phylogenomic study of ST131-H22 from retail poultry meat products and contemporaneous human clinical ST131-H22 isolates in the United States colocalize to the same phylogenetic clade (11), highlighting human-poultry interspecies transmission. However, while evidence exists for and against the hypothesis, the topic remains controversial (18–23).

Unraveling links between human and nonhuman reservoirs of ExPEC is complicated by many factors. Some of these include inherent sampling bias, including overrepresentation of clinical and antimicrobial-resistant (AMR) isolates in public databases. Other issues pertain to nuances of ExPEC phylogenomics and pathobiology, including the delay between colonization of the host gut and disease onset and the genomic diversity and plasticity observed within and between major ExPEC lineages (24). Several key studies that have sought to address spillover between human and animal E. coli reservoirs are limited by sampling based on the selection of isolates that are resistant to critically important antimicrobials (CIAs) (21, 25, 26). Notwithstanding the importance of identifying pathogens resistant to CIAs, viewing microbial communities through an AMR-centric lens potentially obfuscates the timely identification of emerging pathogens and limits understanding of mobile genetic elements (MGEs) that spread virulence and AMR gene cargo (11, 17, 27, 28).

To examine interspecies transmission and foodborne ExPEC infections, studies that focus on microbial transmission between humans, animals, and natural environments coupled with detailed investigations of MGEs that the microbes carry are needed. The ExPEC lineage ST95 is a key candidate for such a study because of the following: (i) it is a leading cause of UTI and bloodstream infection (BSI) (29–31) and a major cause of bacterial meningitis, particularly in newborns (31, 32); (ii) it is infrequently resistant to CIAs despite being pandemic; thus, resistance traits do not explain its global dominance (14); (iii) it is likely to be underestimated as a cause of serious extraintestinal disease (7); (iv) it is often identified as a commensal in the feces of diverse hosts (33–35), so it has a means to spread readily via food and/or water; and (v) it is a likely ExPEC lineage to cause zoonotic infections. Evidence pointing to its candidacy for zoonotic infections includes association of ST95 lineages with infection in both poultry and humans (36) and the carriage of ColV plasmids and other closely related elements such as ColBM plasmids (37), here referred to as ColV-like plasmids (ColVLPs). ColV-like plasmids (i) are definitive of the APEC pathotype (38), (ii) carry an arsenal of virulence genes that encode adhesins, toxins, iron sequestration, and serum survival capabilities critical for survival in extraintestinal sites (39, 40), (iii) are associated with the ability to cause extraintestinal disease in diverse animal models of human disease (41, 42), (iv) play a key role in the evolution of hybrid E. coli pathotypes (43), and (v) confer ExPEC capabilities to otherwise commensal E. coli isolates (41, 44). ColV-associated genes have been also detected in E. coli isolated from retail poultry meat (45, 46).

A study of 86 ST95 isolates sourced from U.S. hospitals identified several major sublineages linked to the carriage of specific *fimH* alleles and examined the genetic basis for the acquisition of antibiotic resistance genes (ARGs) (14). A pansusceptible ST95 lineage carried an F virulence plasmid closely related to pUTI89, whereas lineages that displayed multiple drug resistance (MDR) did not carry such a plasmid (14). pUTI89 and related plasmids carry a suite of virulence genes involved in iron acquisition and toxin production and are required to establish intracellular bacterial communities in bladder epithelial cells (47). Of the 86 isolates in the study, 34 (40%) carried AMR genes, often on large conjugal plasmids that remained largely uncharacterized (14). One of these, pSF-088-1 (48), is a ColV F virulence plasmid that carries a complex resistance region with a unique genetic signature identified in MDR E. coli with diverse STs in Australia and elsewhere (44).

While ColV-like plasmids and virulence plasmids similar to pUTI89 are known to be carried by ST95, plasmid associations within ST95 sublineages are poorly understood. Additionally, genomic epidemiological studies of ST95 to date have not investigated isolates from diverse sources but have compared either ST95 isolates from extraintestinal cases of disease in humans and diseased poultry animals (APEC isolates) (36) or only human-associated isolates (49). To address these gaps, we characterized the genomes of 668 geographically diverse ST95 isolates collected from companion animals, natural environments, food animals, and humans over a 40-year period and applied high-resolution phylogenetic and pangenomic analyses to explore both chromosomal and plasmid lineages. We also screened 34,176 geographically, temporally, and source diverse E. coli genomes comprising 2,570 STs to gain insights into the role of ST95-associated virulence plasmids in ExPEC infections more broadly.

## RESULTS

### Analysis of global, source diverse ST95 isolates revealed a larger pangenome than previously recognized.

**FIG 1:**
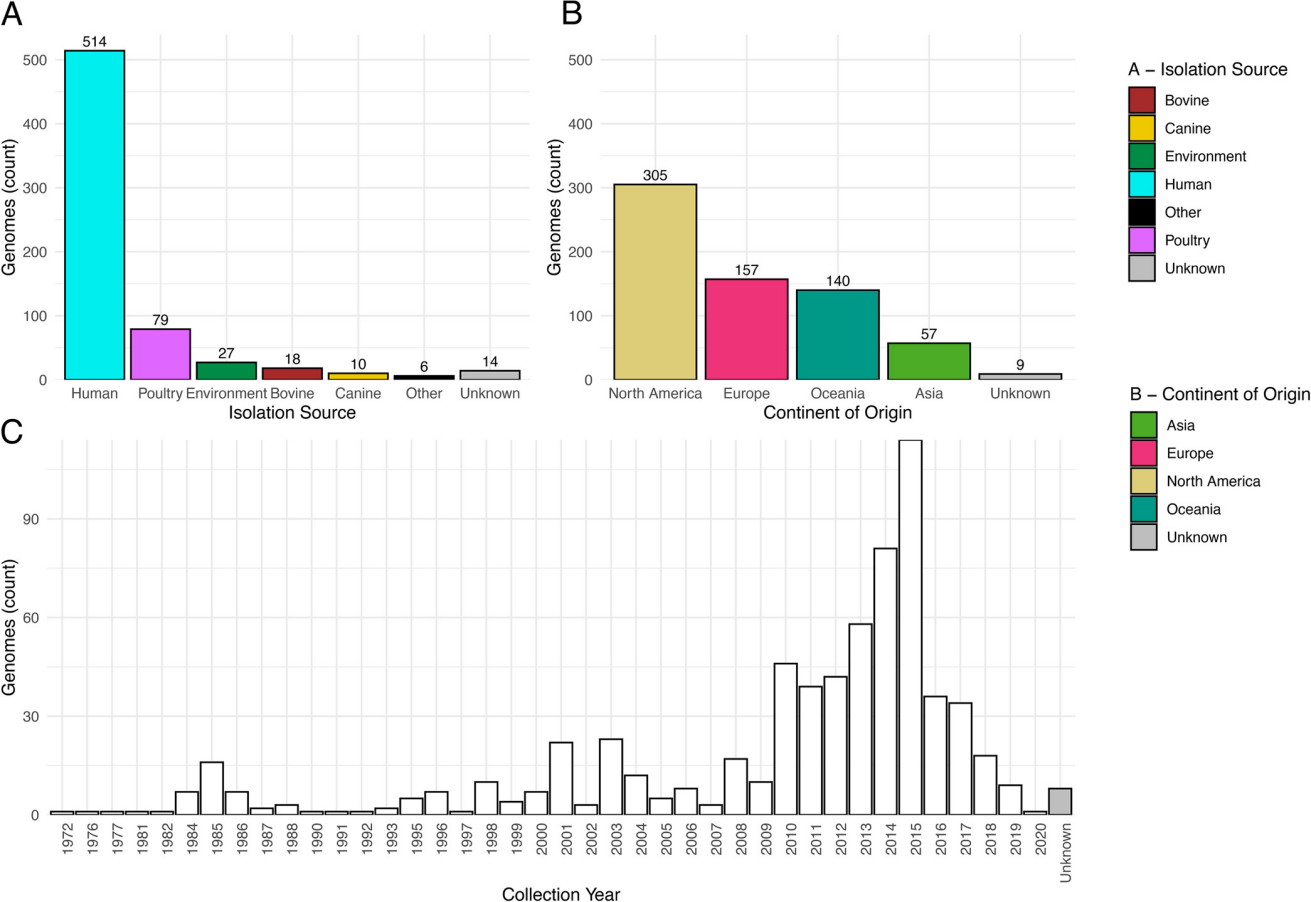
A visualization of the isolation source (A), continent of origin (B), and collection year (C) of 668 ST95 isolates under investigation.

Genomic assemblies of 668 ST95 isolates from diverse niche, geographical, and temporal origins were characterized (Fig. 1). Most originated from humans (514/668 [77%]), of which 74% (380/514) were recovered from patients with a UTI (183/514 [36%]) or a systemic infection (197/514 [38%]). The next largest group of isolates originated from poultry (79/668 [11%]), which included APEC (43/79 [54%]) and isolates sourced from poultry meat (33/79 [42%]). The remaining isolates originated from the environment, cattle, dogs, and other sources; 14 isolates have an unknown origin. Isolates originated across multiple continents and spanned 4 decades (1972 to 2020). Additional metadata are shown in Table S1 in the supplemental material.

10.1128/mSystems.01212-21.2 TABLE S1 Genotypic data and metadata associated with the ST95 cohort. Also shown are Uberstrain numbers for all strains (allowing access via Enterobase) and their NCBI Sequence Read Archive numbers, where applicable. Download Table S1, TXT file, 1.0 MB. Copyright © 2022 Cummins et al. 2022 Cummins et al. https://creativecommons.org/licenses/by/4.0/ This content is distributed under the terms of the Creative Commons Attribution 4.0 International license.

The core and pangenome (which includes both the core and accessory genomes) of ST95 isolates in the collection comprised 3,180 and 22,976 genes, respectively. While the size of the core genome is comparatively stable across studies of ST95 (36, 49, 50), in this study, the pangenome was approximately 50% larger than in previous estimates (36, 49).

### Identification of five novel ST95 clades.

**FIG 2:**
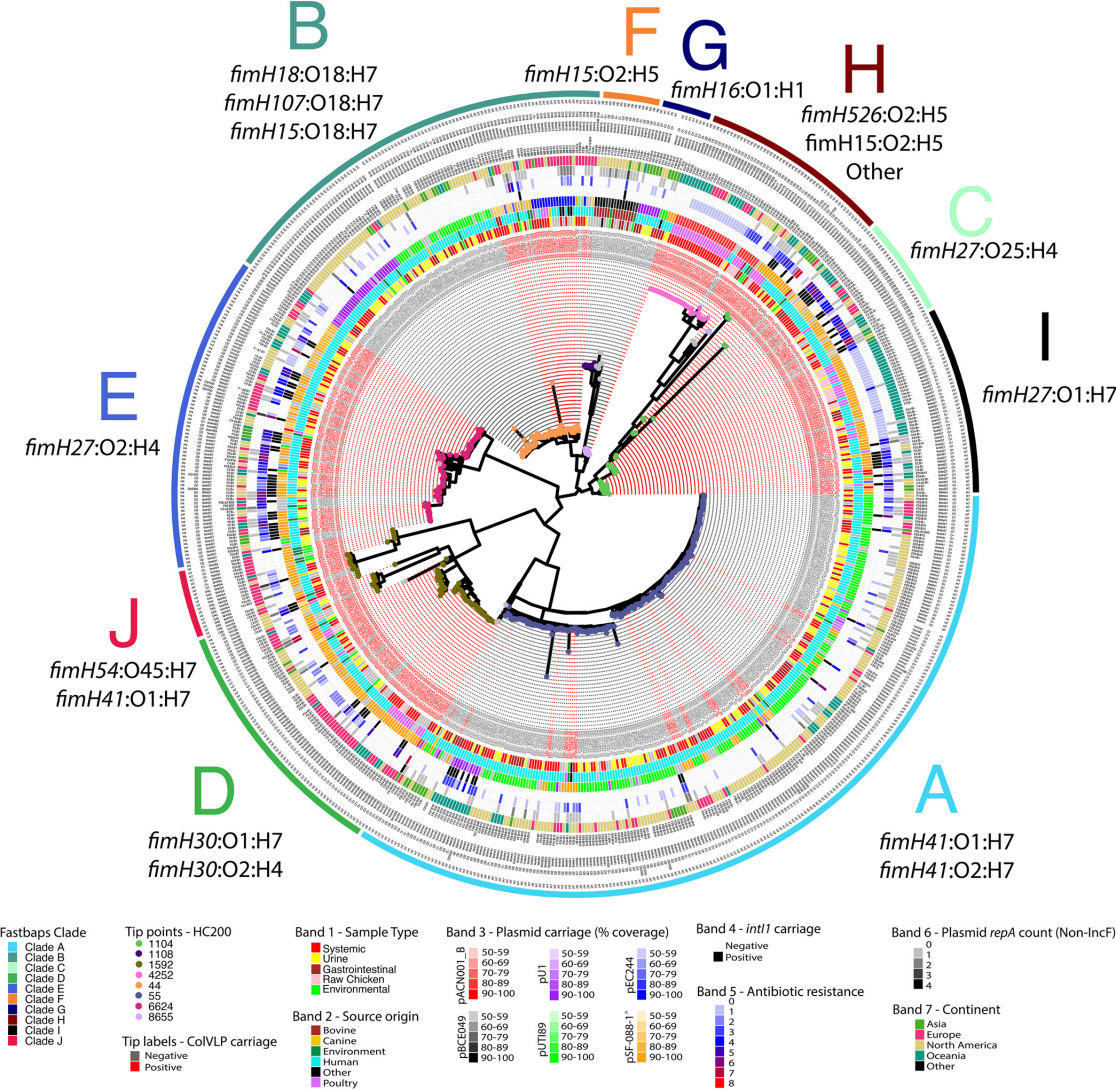
Core genome-based phylogeny visualizing the relatedness of ST95 isolates. Red tip labels (indicating sample names) indicate carriage of a ColVLP plasmid, and gray tip labels indicate no carriage of a ColVLP plasmid (as per the Liu criteria), while tip points are colored based on cgMLST HC200 groups. Concentric colored rings from innermost to outermost show isolate source, coverage of one of six reference plasmids, count of antibiotic classes to which a isolate is resistant, carriage of class 1 integrase gene *intI1*, count of non-IncF *repA* genes, and country of origin. Text outward of these colored bars lists the IncF RST, *fimH* type, O type, and H type. Where a type is missing for a given isolate, F plasmid *repA* genes were not detected. Outermost letters on the core genome phylogeny indicate clade designations, and proximal to a given clade label is the primary *fimH* and serotype(s) associated with a given clade. Tree is midpoint rooted. Detail in this figure is best viewed in its digital format.

Phylogenomic analysis of both the core genome and the accessory genome of ST95 generated trees with similar topologies (Fig. 2 and Fig. S2 [https://github.com/maxlcummins/ST95/blob/main/Supplemental_Fig_S2.tif]) which were in agreement with *fimH* typing, serotyping, fastbaps clade designations, and cgMLST HC200 (see Materials and Methods). In general, isolates of a given HC200 group clustered closely, but within particular HC200 groups some isolates diverged at both core genome and accessory genome levels, such as for isolates of HC200:8655 (Fig. S2 [https://github.com/maxlcummins/ST95/blob/main/Supplemental_Fig_S2.tif]). Our core genome phylogeny in combination with fastbaps identified five previously reported clades of ST95 (A, B, C, D, and E) (50) and an additional five clades (F, G, H, I, and J) (Fig. 2). This clade structure was also supported by an accessory gene phylogeny (Fig. S2 [https://github.com/maxlcummins/ST95/blob/main/Supplemental_Fig_S2.tif]). All clades within this analysis contained isolates sourced from three or more continents, indicating global dissemination.

Clade F, which is predominantly *fimH15* and serotype O2:H5, comprises a predominantly bovine-associated sublineage. Isolates in the closely related clade G are predominantly *fimH16* and serotype O1:H5 and were sourced mostly from humans and carry multiple plasmid types. Clade H is phylogenetically diverse and represents a number of *fimH* types, serotype combinations, and ColV-like plasmids and is enriched with isolates with a poultry source. Isolates in clade I are mostly *fimH27* with serotype O1:H7 and often carry ColV-like plasmids. Lastly, clade J (*fimH54*:O45:H7; previously described as being part of clade D) comprised mostly ColV plasmid-carrying isolates from Asia.

### Evidence of interspecies transmission of E. coli ST95 clones.

**FIG 3:**
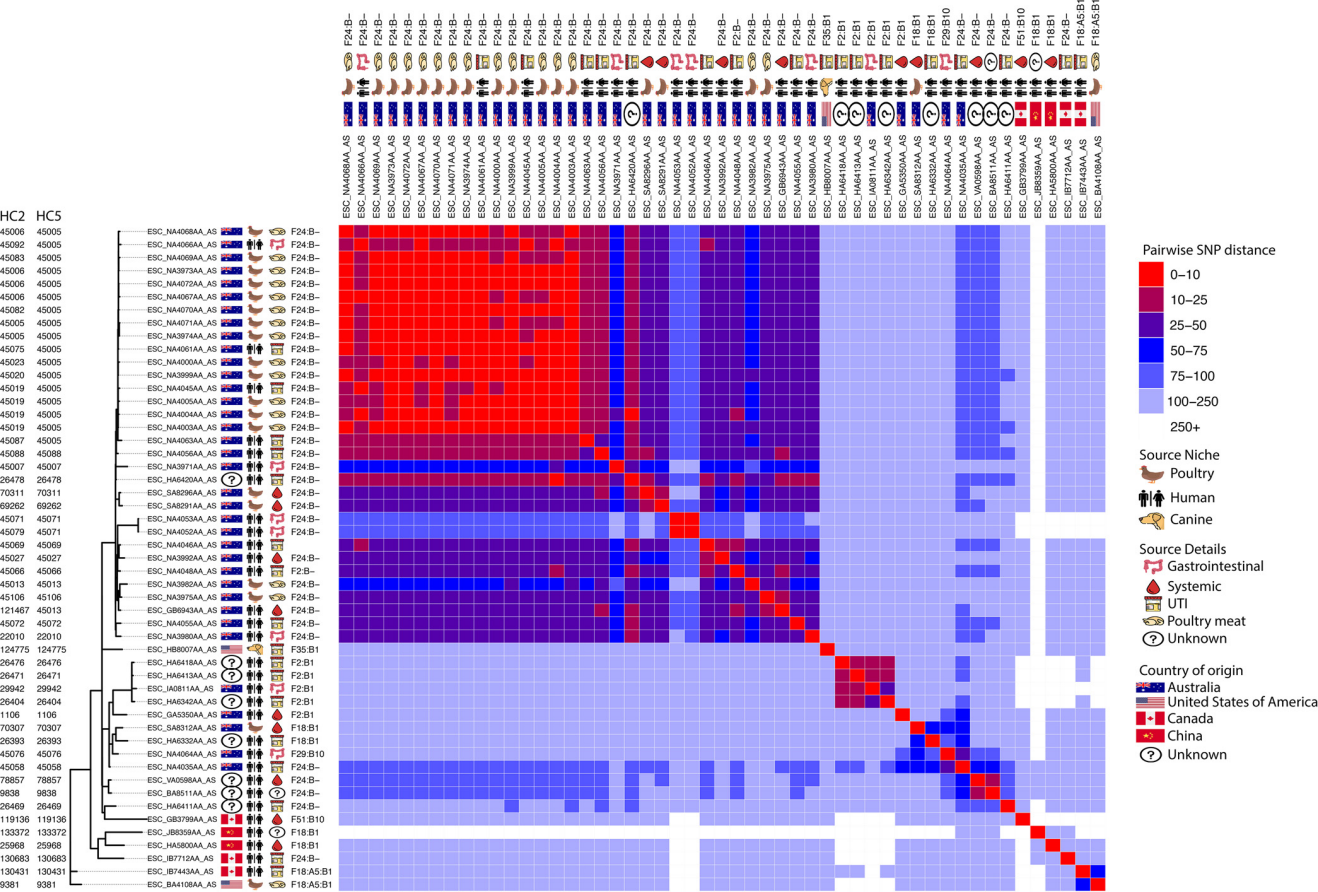
Maximum likelihood tree visualizing the core genome similarity of clade I. Immediately adjacent to tip labels are icons depicting metadata. The portion to the right is a heat map visualizing pairwise SNP distances. Tree is midpoint rooted.

Given the known zoonotic potential of ST95, we examined core genomic differences between pairs of isolates to identify potential interspecies transmission events. Some clades exhibited a degree of host specificity, while others housed isolates from multiple host types (Fig. 2). Intrasource clonality (clonality being defined as isolates exhibiting 25 or fewer core genomic single nucleotide polymorphisms [SNPs]) was observed in multiple clades, while evidence of intersource transmission, particularly between human and poultry sources, was particularly evident in clade I (Fig. 2 and Fig. S3 [https://github.com/maxlcummins/ST95/blob/main/Supplemental_Fig_S3.tif]), highlighting this clade as one of interest in regard to potential zoonotic transfer events. Isolates in clade I originated primarily from Australia (36/51 [71%]), with the remainder being from other (8/51 [16%]) or unknown (7/51 [14%]) continents. Apart from a single isolate from an episode of UTI in a dog from the United States, all were sourced from poultry or humans. Most isolates from poultry were from retail poultry meats, and most human isolates were from extraintestinal infections (Fig. 3). We then characterized clade I isolates in terms of AMR, virulence-associated traits, and F plasmid carriage. Every isolate from clade I carried at least one AMR gene or FQR-associated SNP, but only 24% (12/51) were considered MDR. Notably, 92% of isolates from clade I (47/51) carried tetracycline resistance determinant *tet*(B), but few isolates carried genes conferring resistance to CIAs (Fig. S4 [https://github.com/maxlcummins/ST95/blob/main/Supplemental_Fig_S4.tif]).

Clade I isolates carried the following: iron acquisition genes from the aerobactin (*iucD*) and yersiniabactin (*iroN*) operons (47/51 [92%]), *fecA* (51/51 [100%]), and *fyuA* (51/51 [100%]); genes involved in immune system evasion, including *iss* (51/51 [100%]), *traT* (49/51 [96%]), and *ompT* (49/51 [96%]); neonatal meningitis-associated *E. coli* (NMEC)-associated genes *kpsMTII* (47/51 [92%]), *gimB* (27/51 [93%]), and *neuC* (33/51 [67%]); and the pyelonephritis-associated pilus gene *papGII* (51/51 [100%]). Notably, almost all clade I isolates (46/51 [90%]) carried a ColV-like plasmid (Fig. S4 [https://github.com/maxlcummins/ST95/blob/main/Supplemental_Fig_S4.tif]). Clade I contained an Australian-specific clonal sublineage comprising human ExPEC, human gastrointestinal and poultry meat isolates differing by fewer than 10 SNPs and sharing an HC5 (or in some cases HC2) group, an F replicon sequence type (IncF RST), and high genotypic similarity (Fig. 2 and Fig. S4 [https://github.com/maxlcummins/ST95/blob/main/Supplemental_Fig_S4.tif]). All isolates in this overlap group were sourced from Canberra, Australia, between 2013 and 2016 (46).

### The ST95 F plasmidome is diverse but largely comprises two F virulence plasmid types.

**FIG 4:**
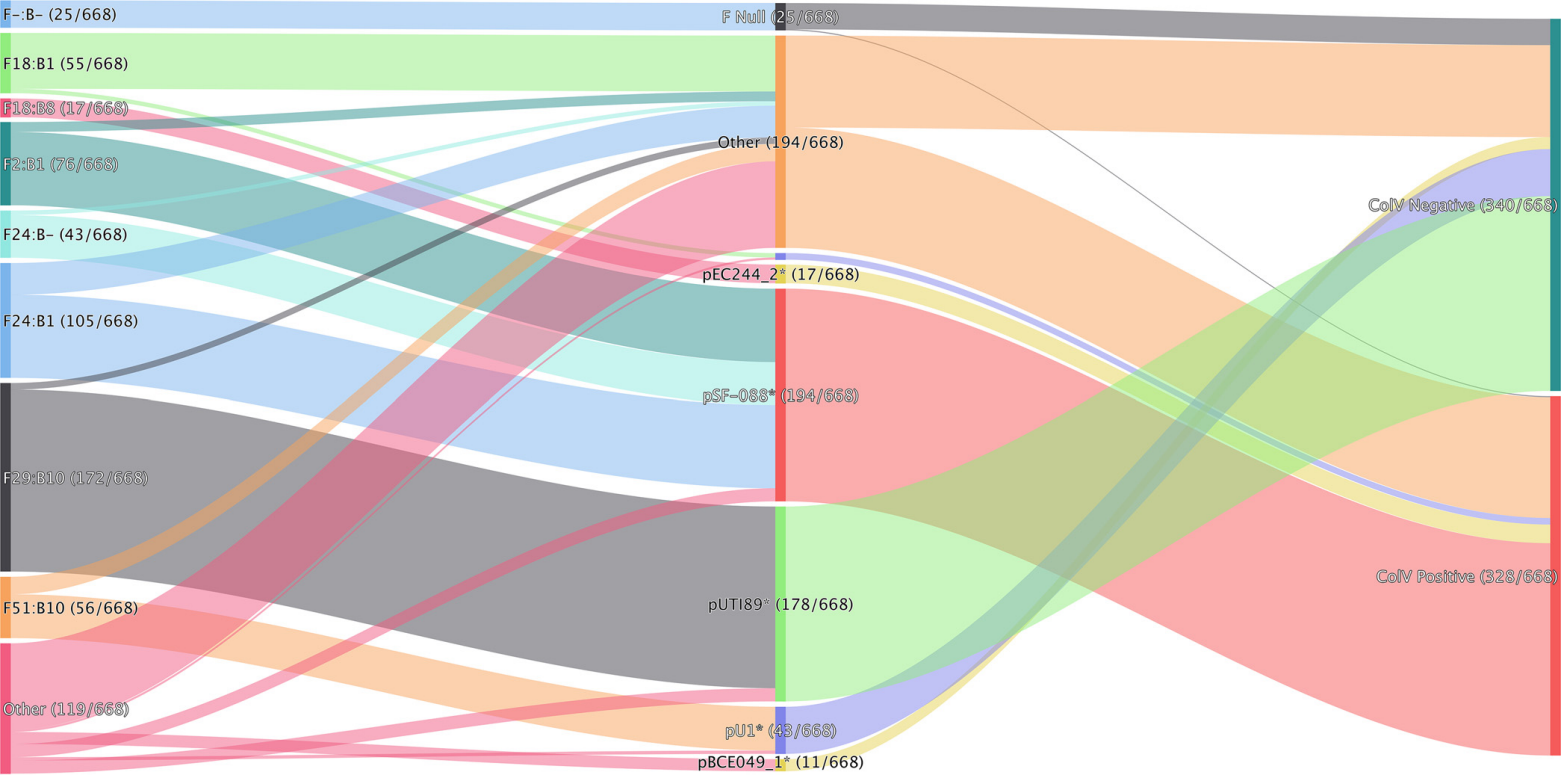
A visualization of the relationship of carriage of reference plasmids, IncF replicon sequence types, and ColV status of ST95 isolate.

We screened the ST95 collection for IncF RSTs, a family of conjugal plasmids known to be common in commensal E. coli and frequent carriers of virulence-associated genes and AMR genes. Our rationale was that this would allow us to detect genetic material with disease-related potential moving between ecological niches. Almost all ST95 isolates (643/668 [96%]) carried an IncF plasmid, as defined by IncF RST (Fig. 4). Two plasmid lineages were substantially more prevalent than others. Of the ST95 isolates carrying an IncF plasmid, approximately half carried ColV-like plasmids (328/668 [49%]). Most (194/328 [59%]) of the ColV-like plasmids were pSF-088-1*, a plasmid type originally isolated from an ST95 sepsis-associated E. coli from San Francisco General Hospital in 2013 (48, 51). Strikingly, more than half (178/340 [52%]) of the remaining ColV-negative isolates harbored pUTI89* (see Materials and Methods for explanation of asterisk) instead. pUTI89 (IncF RST F29:B10) is a well-characterized plasmid with roles in UTI pathogenesis (47, 52). It was originally described for a uropathogenic E. coli (UPEC) isolate, UTI89 (ST95:O18:K1:H7) (53), and is frequently encountered in clinical E. coli isolates (47, 54). In our analysis, we found that isolates carrying ColV-like plasmids carried significantly more virulence-associated genes than their counterparts that did not carry such plasmids (mean counts of 49.4 and 44.4, respectively [*t* test *P* ≤ 2.2e−16]) (Table S1).

pSF-088-1* plasmids, typical of many of the ColV plasmids in our ST95 collection, were present in ST95 isolates from both human-associated (133/514 [26%]) and poultry-associated (41/79 [52%]) ST95 isolates. In contrast, pUTI89* plasmids were niche restricted. Ninety-nine percent of pUTI89* plasmids carrying ST95 originated from humans (176/178). This plasmid was absent from poultry isolates and was present in nonhuman sources in only 1% of cases (2/178). To a lesser extent, other ColV-like plasmids were observed in our collection, as were other less predominant non-ColV-like plasmids (Fig. 4 and Table S1).

### pUTI89 and ColV-like plasmids are common in pandemic ExPEC lineages.

We sought to contextualize F plasmid carriage within the broader context of E. coli. To this end, we analyzed IncF RSTs associated with pUTI89* and ColV-like plasmids among 34,176 genomic assemblies from Enterobase comprising 2,570 multilocus sequence types (MLSTs). The patterns of prevalence in the wider E. coli set were broadly similar to those seen in our ST95 collection. Likewise, in the wider E. coli set, the association between plasmid lineage and host range mirrored that seen in our ST95 collection.

**FIG 5:**
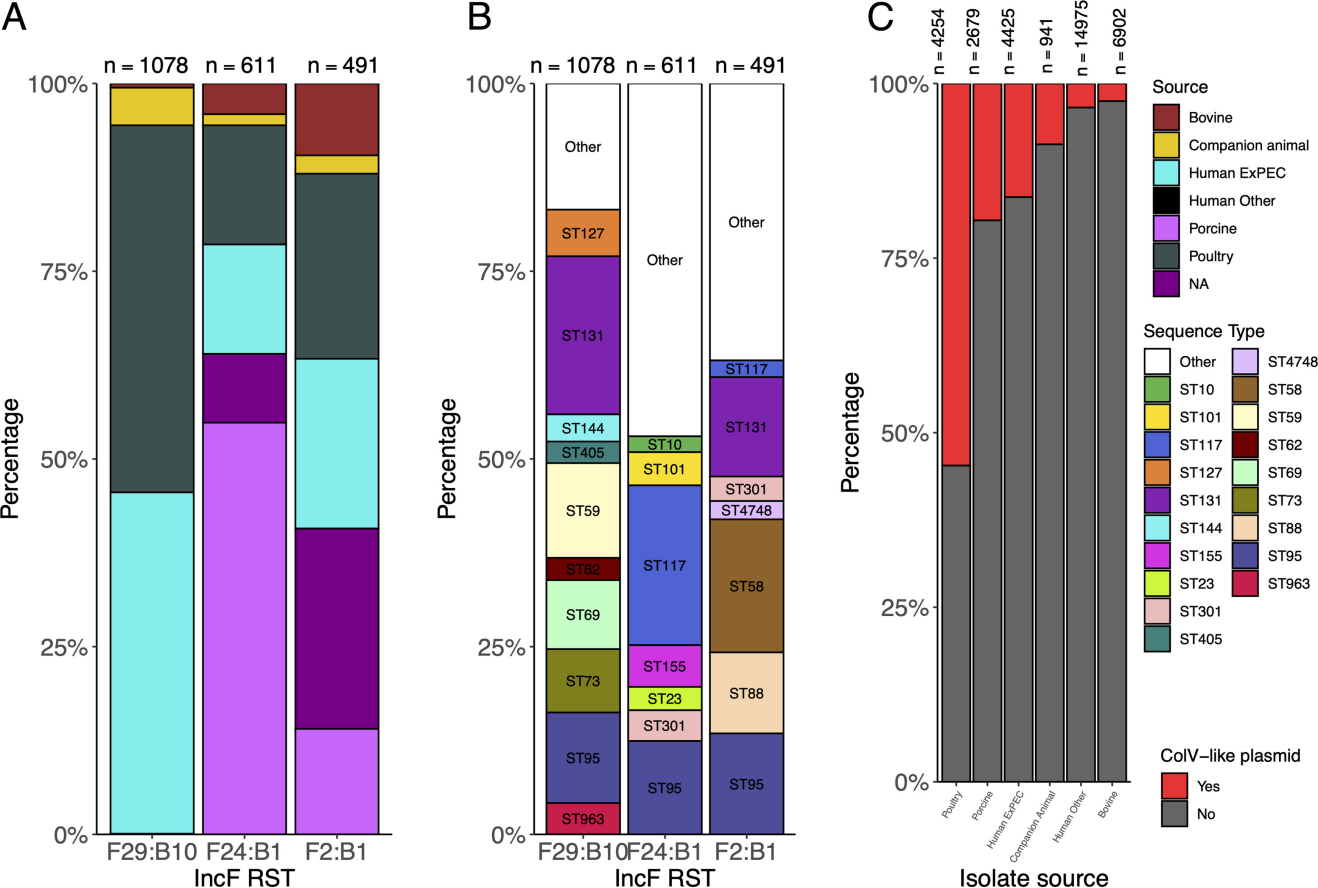
Plasmids and sequence types present in 34,176 genomes from Enterobase. (A) Distribution of source associations among IncF RST-carrying isolates; (B) distribution of STs among IncF RST-carrying isolates; (C) ColV-like carriage versus source association of E. coli.

F29:B10 (the IncF RST typical of pUTI89) was the third most prevalent IncF RST across all E. coli isolates (Fig. 5). Approximately 94% of genomes with F29:B10 were sourced from humans, more than half of which were sourced from humans with extraintestinal disease. Furthermore, while F29:B10 was the second most common IncF RST in humans, it was notably absent from the 4,345 poultry isolates available for analysis and almost completely absent from E. coli sourced from pigs (1/2,679 [0.04%]) and cattle (6/6,902 [0.09%]) (Fig. 5A). ColV-like plasmids with IncF RSTs F24:B1 and F2:B1 appeared frequently in the ST95 cohort in poultry-associated and human ExPEC isolates, and this observation was mirrored in the broader E. coli cohort (Fig. 5).

Our analysis of the intersection of E. coli MLST and IncF RST found that F29:B10-associated STs include major pandemic ExPEC lineages such as ST131, ST73, ST69, and ST95, but the association was source restricted within those STs. We also found that ColV-like plasmids were highly represented among poultry isolates (2,327/4,254 [55%]) but also present in 16% (720/4,425) of human ExPEC isolates. A complete list of isolate metadata and IncF RSTs are available in Table S2 (https://github.com/maxlcummins/ST95/blob/main/Supplemental_Table_S2.txt).

### Multidrug resistant ST95 isolates are associated with carriage of ColV-like plasmids and a class 1 integrase.

Twenty-two percent (148/668) of isolates in the ST95 cohort were resistant to three or more antibiotic classes based on genotype, and nearly half (320/668 [48%]) carried one or more antibiotic resistance genes (Table 1). Most prevalent were genes (or SNPs) associated with resistance to β-lactams, trimethoprim/sulfonamides, aminoglycosides, and fluoroquinolones. Infrequently encountered were genes encoding resistance to CIAs.

**TABLE 1:** Genotypic resistance among ST95 isolates and its relationship with carriage of ColV-like plasmids

Antimicrobial class or resistance type	No. (%)		*P* value^*a*^	Total count, no. (%) (*n* = 668)
	ColV^+^ (*n* = 328)	ColV^−^ (*n* = 340)		
Beta-lactams	109 (33.2)	80 (23.5)	**	189 (28.30)
Sulfonamides	122 (37.2)	49 (14.4)	***	171 (25.60)
Aminoglycosides	93 (28.4)	51 (15.0)	***	143 (21.40)
Tetracyclines	105 (32.0)	17 (5.0)	***	122 (18.30)
Trimethoprim	87 (26.5)	21 (6.2)	***	108 (16.20)
Fluoroquinolones^*b*^	44 (13.4)	22 (6.5)	**	66 (9.90)
ESBLs^*b*^	22 (6.7)	15 (4.4)	-	37 (5.50)
Amphenicols	16 (4.9)	4 (1.2)	**	20 (3.00)
Macrolides/lincosamides^*b*^	10 (3.0)	8 (2.4)	-	18 (2.70)
Polymyxins^*b*^	3 (0.9)	0 (0.0)	-	3 (0.45)
AMR	216 (65.9)	104 (30.6)	***	320 (47.90)
MDR	99 (30.2)	49 (14.4)	***	148 (22.20)
CIA resistance	62 (18.9)	35 (10.3)	***	97 (14.50)

a ***, *P* ≤ 0.0005; **, *P* ≤ 0.005; *, *P* ≤ 0.05; -, *P* > 0.05.

b Critically important antimicrobials.

An investigation of plasmid type and resistance gene carriage (Table 1) revealed a positive correlation between AMR gene and ColV-like plasmid carriage. Of the 320 ST95 isolates in our cohort with one or more antibiotic resistance genes, 68% (216/320) carried ColV-like plasmids (χ^2^ test *P* ≤ 2.2e−16) (Table S3). Similarly, of the 148 MDR ST95 isolates in our cohort, 67% (99/148) carried ColV-like plasmids (χ^2^ test *P* = 9.259e−07). Isolates carrying ColV-like plasmids exhibited higher genotypic resistance (average of 1.8 classes of antibiotics per isolate) than ST95 isolates that did not carry ColV-like plasmids (average of 0.74 class of antibiotic per isolate) (*t* test *P* = 6.8e−15). Notably, AMR and MDR among ColV-positive isolates was not class specific for any antibiotic. Genotypic resistance was significantly more common among ColV^+^ ST95 isolates for almost all classes of antibiotics investigated (Table S3), with genes encoding ESBLs, macrolides/lincosamides, and polymyxins an exception. It was notable but perhaps unsurprising that AMR (χ^2^ test *P* ≤ 2.2e−16) and MDR (χ^2^ test *P* ≤ 2.2e−16) isolates were significantly associated with carriage of the class 1 integrase gene *intI1*, a gene significantly more common in isolates with ColV-like plasmids (Table S3).

10.1128/mSystems.01212-21.3 TABLE S3 Associations of plasmid carriage and resistance traits in ST95. Shown are the relative proportions of genotypic antimicrobial resistance, genotypic multidrug resistance, plasmid carriage (non-IncF *repA* genes), and *intI1* carriage for strains carrying different plasmid types. Also listed are the associations of *intI1* with genotypic antimicrobial resistance and genotypic multidrug resistance. Download Table S3, XLSX file, 0.01 MB. Copyright © 2022 Cummins et al. 2022 Cummins et al. https://creativecommons.org/licenses/by/4.0/ This content is distributed under the terms of the Creative Commons Attribution 4.0 International license.

Given the prevalence of pUTI89* among ColV^−^ isolates, we also investigated the association between pUTI89* carriage and resistance traits. pUTI89*-carrying isolates exhibited less carriage of AMR genes (measured as the count of classes to which an isolate carried an AMR gene) than the broader ST95 cohort (0.39 versus 1.26 [*t* test, *P* = 6.142e−16]) but also relative to other ST95 isolates which were ColV^−^ but carried other F plasmid types (0.39 versus 0.74; *P* = 0.001541). In summary, ST95 isolates that carry pUTI89* carry significantly fewer AMR genes than other ST95 isolates irrespective of their ColV plasmid status.

## DISCUSSION

### Clonal overlap of ST95 isolates from poultry and humans indicates interspecies transfer.

A large body of *in vivo*, *in vitro*, and *in silico* evidence now substantiates the zoonotic capacity of ExPEC and APEC lineages and the potential occurrence of foodborne UTIs (1, 16, 18, 36, 55). However, examples of clonally related isolates from poultry and humans with epidemiologically relevant geographical and temporal linkage are limited, and as a result, the foodborne-UTI hypothesis remains controversial. Our analysis of ST95, particularly the clonal poultry-human group identified in clade I, adds considerable weight to the zoonotic capacity of ExPEC and APEC lineages and the foodborne-UTI hypothesis. The extensive similarity in the core and accessory genomes of clade I ST95 isolates, along with their tight temporal and geographical linkage, strongly suggests multiple transfer events between poultry and humans. In addition, we note that our observations from analyses of clade I would not have come to light if selection of ST95 isolates for this study had been prefaced on nonsusceptibility to CIAs. In addition, our data support the contention that colocalized and time-sensitive, cross-source sampling is invaluable in investigating the potential for interspecies transmission of ExPEC pathogens (23).

The directionality of interspecies transmission is challenging to predict, and we also cannot rule out coacquisition of these clones from a so-far-uncharacterized source. Nonetheless, the weight of evidence suggests that the flow is from poultry to humans. Worldwide, there are approximately 26 billion chickens (56), and colibacillosis caused by APEC that overwhelmingly carry ColV plasmids is the most common bacterial poultry disease (57). Poultry-associated E. coli (i) are common as contaminants of retail poultry meat (46, 58), (ii) contaminate areas used to prepare poultry meats (59, 60), and (iii) are recovered from the gastrointestinal tracts of healthy humans (61). Furthermore, demand for poultry products is increasing (62, 63), and poultry manure is used widely as fertilizer, despite being rich in AMR and pathogenic E. coli (64). Poultry consumption is also associated with an increased risk of contracting an antibiotic-resistant UTI (65). Collectively, these data support the contentions that poultry constitute a reservoir of human ExPEC, that poultry products may constitute a means for the dissemination of these pathogens, and that the occurrence of such dissemination events might be expected to increase as the poultry sector grows.

It is also notable that nearly all members of the poultry-human overlap group (50/51) in clade I carry ColV-like plasmids. While ColV-like plasmids are common in many sources, including swine, they are disproportionately carried by poultry-associated E. coli (Fig. 5). Isolates hosting ColV-like plasmids exhibit enhanced ability to form biofilms (66) and display resistance to chlorine (67). Chlorine is used in some countries to control microbial contamination of freshly slaughtered poultry, and isolates carrying ColV plasmids may have a competitive advantage for persistence on poultry meats (68).

### ColV-like plasmids are common in ST95 isolates, linked with broad host range and pathogenicity, and linked with increased AMR and virulence.

Beyond clade I, analysis of the plasmidome of our ST95 collection identified ColV-like plasmids as prevalent among both poultry and human isolates. All of the ST95 isolates sourced from poultry carried a ColV plasmid (79/79), including poultry meat and APEC, as did 42% (216/514) of human isolates, 25% (18/72) of gastrointestinal E. coli isolates, and 41% (156/380) of human ExPEC isolates. This multihost distribution in both commensal and pathogenic niches can be explained by the fact that ColV-like plasmids carry genes which can enhance gastrointestinal colonization as well as extraintestinal pathogenicity in both poultry and humans (41, 47, 69–71).

Carriage of ColV-like plasmids was associated with significantly higher counts of virulence-associated genes. This is expected; however, our results also suggest that the contribution to virulence of ST95 made by ColV-like plasmids may be accelerated through antibiotic selection pressures coselecting for more virulent E. coli lineages. In our study, isolates carrying ColV-like plasmids, on average, carried more resistance genes than their counterparts without such plasmids. Further, AMR genes could be linked to scaffolds containing fragments of ColV plasmids in ST95 assemblies (Table S4). These observations are in line with previous studies showing that some ColV-like plasmids carry virulence and resistance gene cargo (17, 35, 40, 44, 48). We also observed shared IncF RSTs across ST95 isolates from different sources and across phylogenetic backgrounds, suggesting intersource plasmid transmission events. These findings are also consistent with those of other studies showing that closely related plasmids are shared between humans, food animals, and urban-adapted wild birds (12, 17, 72, 73).

10.1128/mSystems.01212-21.4 TABLE S4 Colocalized AMR and IncF *repA* genes among ST95 assemblies Table S4, TXT file, 0.02 MB. Copyright © 2022 Cummins et al. 2022 Cummins et al. https://creativecommons.org/licenses/by/4.0/ This content is distributed under the terms of the Creative Commons Attribution 4.0 International license.

Given the scale of global poultry production and the dominance of E. coli lineages such as ST95, ST117, ST73, ST350, ST429, ST57, and ST973, many of which carry ColV-like plasmids with class 1 integrons (37), poultry represent a huge reservoir for these F virulence plasmids. Stewardship practice in Australia has likely played a major role in preventing the introduction of genes encoding resistance to CIAs from becoming endemic in intensive animal industries (37, 74), but the same cannot be said for poultry operations overseas (75–77). Our analysis did detect the presence of the insertion element IS*26* on scaffolds derived from ColV-like plasmids in ST95 isolates in our collection. IS*26* is an element renowned for capturing and mobilizing ARGs (73, 78), assembling complex resistance regions (79), promoting plasmid fusion (80, 81), and ameliorating plasmid fitness cost (81). This has important implications, as it increases the likelihood of these variants capturing further and novel AMR gene cargo, including genes encoding resistance to CIAs.

### pUTI89 carriage is associated with low AMR in ST95 and associated with human sources across diverse E. coli sequence types.

Carriage of pUTI89* was notable in the global ST95 cohort. Stephens et al. described carriage of pUTI89-like plasmids by a human-centric cohort of ST95 as being strongly associated with pansusceptibility to antibiotics (14). The results of our more expansive study are consistent with this earlier observation. These authors posited that carriage of pUTI89* in E. coli potentially blocks acquisition of other non-IncF plasmids (14), which may in part explain this phenomenon. On a cautionary note, our data do not rule out the possibility that isolates carrying pUTI89* plasmids may acquire antibiotic resistance genes, a notion supported by the observation that pUTI89 and some highly related plasmids carry a copy of IS*26*. Consistent with this view, we identified multiple instances of carriage of multiple resistance genes in isolates carrying pUTI89* among the 34,176 E. coli sequences from Enterobase (data not shown).

It should be emphasized that pUTI89* has been identified in many of the major pandemic ExPEC lineages, including ST95, ST131 (11, 82), and ST73 (83). Unlike ColV-like plasmids, carriage of pUTI89* is indicative of the host range of ST95 and is strongly associated with human ExPEC but entirely absent from isolates in our ST95 collection sourced from poultry (Fig. 2 and 5). Similarly, carriage of pUTI89-associated IncF RST (F29:B10) from 34,176 E. coli isolates from Enterobase indicates that it is virtually absent from E. coli circulating in animal reservoirs, including poultry, porcine, and bovine isolates. Our data therefore provide evidence that carriage of pUTI89* plasmids may be a hallmark of human-associated E. coli lineages irrespective of phylogenetic background. Genetic elements on pUTI89 may incur a fitness cost for E. coli and poorly colonize nonhuman hosts. Further work is needed to investigate this hypothesis. Improved understanding of genes carried on the pUTI89 backbone, their biological relevance, and their conservation in other closely related mobile genetic elements is scope for further study. Such knowledge could be used to develop a screening method for pUTI89* plasmids, similar to the criteria of Liu et al. for ColV carriage (11) that were applied with great success in this study.

Our study provides novel insight into the phylogenetic structure of ST95 and its interplay with F plasmid carriage, antibiotic resistance, and host range. Our work (i) focuses on the clinical importance of ST95 and other ExPEC that carry pUTI89* (IncF-RST F29:B10) and their restricted colonization of humans irrespective of phylogenetic background and (ii) highlights the potential for emergence of variants of this plasmid lineage that carry antibiotic resistance genes in what is otherwise a lineage of virulence plasmids with very minimal carriage of antibiotic resistance genes. Our observations in part provide some insight toward understanding why interspecies transmission of pathogenic E. coli between humans and food animals is complex (84) and needs to be addressed by avoiding sampling regimes underpinned by resistance to CIAs. We also cast further light on the important role of ColV-like plasmids in extraintestinal disease, their preponderance in E. coli lineages that colonize intensive animal production systems, particularly poultry, and their propensity to readily acquire antibiotic resistance genes (35, 44, 61). Finally, we provide some of the most compelling genomic epidemiological evidence thus far for the occurrence of foodborne UTIs caused by ST95 and profile this sequence type as a case study for One Health investigations into the host origins of ExPEC.

## MATERIALS AND METHODS

### Documentation and reproducibility.

For the purpose of *in silico* reproducibility, a GitHub repository available at https://github.com/maxlcummins/ST95 provides the information and files necessary to perform all computational analysis, postprocessing, and data visualization steps, save for minor figure annotations performed in an image editor.

### Genome origins and isolate selection and data access.

Genomic assemblies of ST95 isolates were identified using Enterobase’s (85) built-in search functionality. Note that no selection criteria for AMR isolates were employed. Enterobase was used to download these genomic assemblies and to source core genome MLST (cgMLST), serotype, and *fimH* type data (86). Strains with missing values for year, source, or country of isolation were removed. We also required genomic assemblies to have 50,000 or fewer low-quality bases, contain 600 or fewer scaffolds, exhibit genome sizes between 4.5 Mbp and 6.5 Mbp, and have a minimum coverage of 20. Accession numbers, metadata, and genotypic data are available for the ST95 cohort in Table S1.

An additional cohort of 34,176 phylogenetically diverse E. coli genomic assemblies comprising 2,570 STs was also downloaded from the same source. For accession numbers and metadata for this broader cohort, please see Table S2 (https://github.com/maxlcummins/ST95/blob/main/Supplemental_Table_S2.txt). Note that while Enterobase houses considerably more genomes than we have downloaded, many genomes were excluded due to lacking metadata or having insufficient assembly quality. Scripts detailing the metadata and quality requirements can be found at https://github.com/maxlcummins/ST95.

### Core genome multilocus sequence typing, pangenome-based phylogenetic analysis, and tree visualization.

Genomes were annotated with dfast (87) version 1.2.13 using the option “–use_locustag_as_gene_id,” while pangenomic analysis was performed using Roary (88) version 3.12.0 with the command line options “-e -n -r.” This facilitated the generation of a reference-independent ST95 phylogeny, a methodology chosen due to reference biases and their limited utility in quantifying SNP distances between isolates which are not clonally related. This involved creating a core genomic alignment for all ST95 isolates using Roary, totaling 2,953,300 bp in length. Subsequently, 17,234 variable sites were extracted from this alignment using *SNP-sites* version 2.4.1 (89), and maximum likelihood phylogenetic trees were built using IQtree version 2.0.3 (90) with ModelFinder (91) and UFBoot (92) enabled (options “-m MFP -bb 1000”), resulting in selection of the “GTR+F+ASC+R2” model and 1,000 bootstrap replicates. Pairwise SNP distances between isolates were quantified from the variable-site alignment using snp-dists (https://github.com/tseemann/snp-dists). Clade designations for tips were performed using the clustering algorithm fastbaps (93) version 1.0.4 with the variable-site alignment and its associated tree using the option “-pp baps.”

This process, save for fastbaps designation, was performed again for isolates from clade I to increase the size of the core genome and therefore provide greater confidence in the pairwise SNP distances between clade I isolates. For the second core genomic alignment, a 3,788,080-bp core gene alignment was generated, from which 1,432 variable sites were identified and used to generate a maximum likelihood tree with IQtree using the “K3P+ASC+R3” model and 1,000 bootstrap replicates.

To support data on core genomic SNP distances, we also utilized cgMLST and cgMLST hierarchical clustering (HCC). cgMLST clusters genomes based on their carriage of allelic variants of 2,350 core genomic loci. Enterobase’s cgMLST database allows for grouping of isolates into hierarchical clusters which provide various levels of phylogenetic granularity based on core genome similarity (HC0 up to HC2350). Genomes sharing an HC2 level differ by ≤2 genes in the cgMLST scheme, for example. More information is available at https://enterobase.readthedocs.io/en/latest/features/clustering.html. Note that the ST95 core genome generated in the process described above is a different core genome from that used in the cgMLST scheme.

Phylogenetic trees and SNP distance matrices were visualized using custom R (94) scripts (https://github.com/maxlcummins/ST95) utilizing R packages ggtree 1.16.6 (95), dplyr 0.8.3 (96), magrittr 1.5 (97), ggplot2 3.2.1 (98), readr 1.3.1 (99), reshape2 1.4.3 (100), tidytree 0.2.6 (101), and ggimage 0.2.8 (102). For more detailed methodology, please visit https://github.com/maxlcummins/ST95.

### Genotypic characterization.

AMR-associated point mutations were detected using Pointfinder (103), while genotyping was performed by abricate version 0.9.8 (https://github.com/tseemann/abricate). Resistance, virulence, insertion sequence (IS), and plasmid gene databases utilized include CARD (104), virulence factor database (105), ISFinder (106) (https://www-is.biotoul.fr), and PlasmidFinder (107), respectively. Custom databases were used to genotype isolates for carriage of ColV-like plasmids as per the criteria of Liu et al. (11) and determine carriage of genes of interest absent in the aforementioned databases. All databases utilized are available at https://github.com/maxlcummins/ST95/dbs/abricate. The script used to process abricate, Pointfinder, IncF RST, and ColVLP determination data is also available therein.

Please note that here we define (i) resistance to a particular class of antibiotic as carriage of a gene or SNP associated with resistance to that class of antibiotic, (ii) AMR as carrying one or more genes or SNPs associated with resistance to one or more classes of antibiotic, (iii) MDR as carrying genes or SNPs associated with resistance to three or more classes of antibiotic, and (iv) critically important antimicrobial (CIA) resistance as carriage of genes conferring resistance to the highest-priority CIAs as defined by the WHO (108).

### Plasmid screening and ColVLP determination.

Multiple approaches were used to explore the plasmid lineages within the ST95 cohort, including IncF RST (109), determination of ColVLP-carriage using the criteria of Liu et al. (11), and alignment to determine assembly coverage across reference plasmids. The last approach was utilized to cluster plasmid types across ST95 isolates with various IncF RSTs but similar plasmid backbones. Reference plasmids were selected by screening for the IncF RSTs of isolates, identifying representatives of major IncF RSTs, and manual extraction of scaffolds containing IncF *repA* genes was undertaken prior to querying NCBI’s nucleotide database for complete plasmid sequences. Best-match reference plasmids were then downloaded and used to generate BLAST databases. Reference plasmids include pSF-088-1, pUTI89, pBCE049, pU1, pEC244, and pACN001_B (GenBank accession numbers CP012636, NC_007941, CP042247, CP041358, CP019018, and KC853435, respectively). The relatedness of these plasmids can be seen in Fig. S1. Note that pSF-088-1 contained a resistance region which was removed to ensure that isolates carrying plasmids with close homology to the pSF-088-1 backbone were not penalized for absent resistance loci. This also allows a fairer comparison with the other reference plasmids which do not carry resistance loci and prevents isolates meeting coverage thresholds due to large AMR-associated MGEs present in other genetic contexts. Fasta files used to create databases for reference plasmid screening are available at https://github.com/maxlcummins/ST95/dbs/abricate.

10.1128/mSystems.01212-21.1 FIG S1 A visualization of relatedness for reference plasmids compared with ColV plasmid pSF-088. Note that the resistance region has been removed from the reference plasmid, as described in Materials and Methods in the main text, to allow fairer calculations of plasmid coverage in comparison with other reference plasmids shown. Labeled virulence genes are those which are included in the ColV-like plasmid typing scheme of Liu et al. Download FIG S1, TIF file, 0.5 MB. Copyright © 2022 Cummins et al. 2022 Cummins et al. https://creativecommons.org/licenses/by/4.0/ This content is distributed under the terms of the Creative Commons Attribution 4.0 International license.

Subsequently, nucleotide databases were generated for each database using BLASTn function “makeblastdb” with the option “-hash_index.” Abricate was then used to screen for carriage of a given plasmid, and isolates were defined as carrying plasmids closely related to a given reference where they shared ≥ 95% nucleotide identity and ≥ 90% coverage with a given reference plasmid. Note that for ease of writing, a given reference plasmid match is noted by using reference plasmid’s name followed by an asterisk (e.g., pUTI89* carriage denotes where a genome exhibited ≥ 95% nucleotide identity and ≥ 90 coverage of pUTI89). In regard to the criteria of Liu et al. for ColVLP carriage, genomes exhibiting carriage of one or more ColV genes (defined as ≥95% nucleotide identity and ≥ 90% coverage) from four or more of the following sets of genes were considered to carry a ColVLP: (i) *cvaABC* and *cvi* (the ColV operon), (ii) *iroBCDEN* (salmochelin operon), (iii) *iucABCD* and *iutA* (the aerobactin operon), (iv) *etsABC*, (v) *ompT* and *hylF*, and (vi) *sitABCD*. Custom scripts used to compute reference plasmid coverage, determine ColVLP carriage, and process IncF RST data are available at https://github.com/maxlcummins/ST95.

### Statistical analysis.

Comparisons of the frequencies of traits between groups of isolates, such as AMR and MDR rates among isolates with or without ColV-like plasmid carriage, were performed using the chi-square test. Comparisons of trait mean differences between groups of isolates were performed with a two-tailed Student *t* test. For both tests, a significance cutoff (*P*) of ≤0.05 was utilized. See https://github.com/maxlcummins/ST95 for scripts detailing statistical analysis.

### Data availability.

We provide the SRA accession numbers for all genomic data sets deposited in NCBI’s Sequence Read Archive (SRA) in Tables S1 and S2. For genomic assemblies produced by reads directly uploaded to Enterobase, assembly numbers are available in the same source. No additional genomic data were uploaded to either Enterobase or SRA by us for the purpose of this study.
