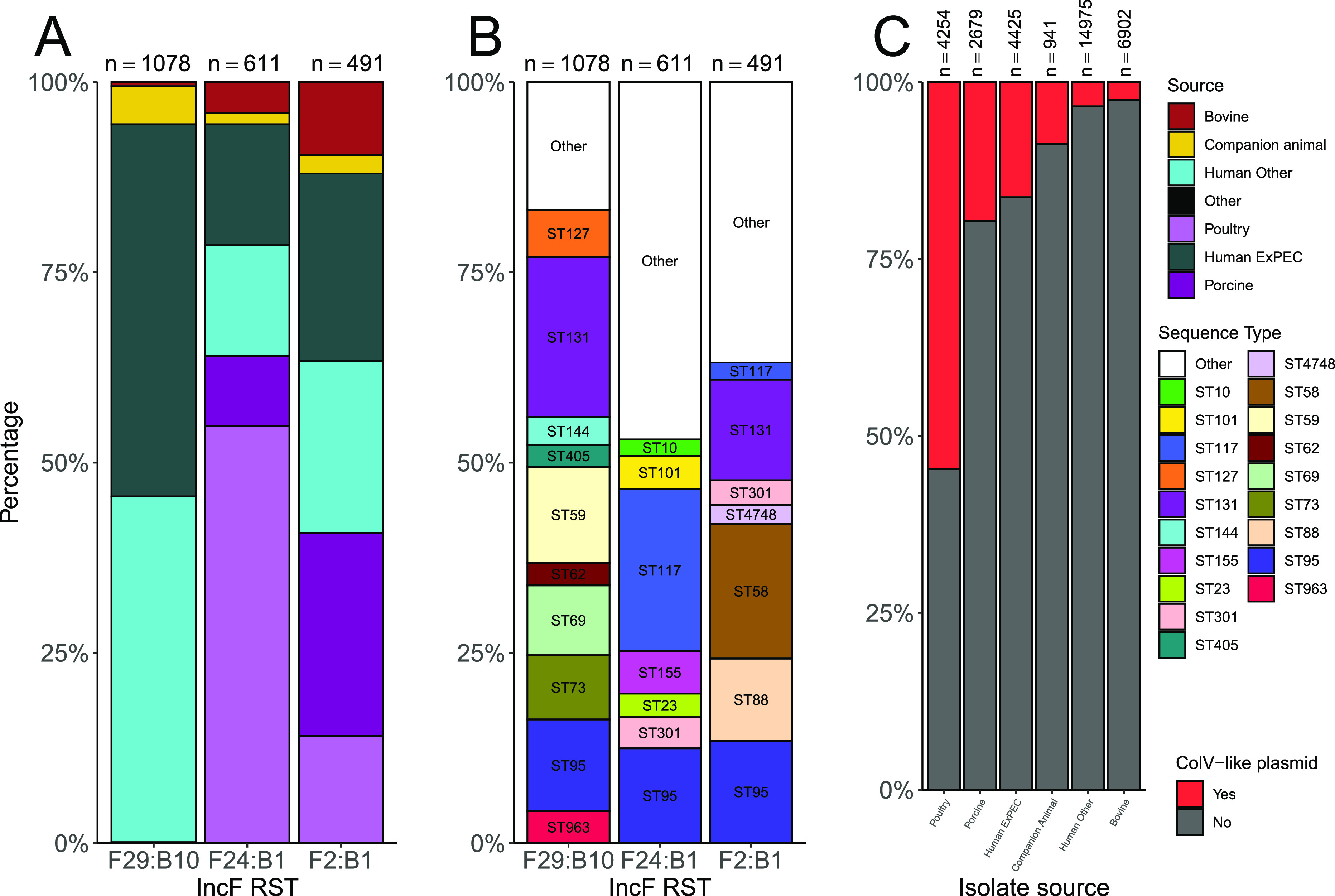# Erratum for Cummins et al., “F Plasmid Lineages in Escherichia coli ST95: Implications for Host Range, Antibiotic Resistance, and Zoonoses”

**DOI:** 10.1128/msystems.00210-22

**Published:** 2022-03-21

**Authors:** Max Laurence Cummins, Cameron J. Reid, Steven Philip Djordjevic

**Affiliations:** a The iThree Institute, University of Technology Sydney, Ultimo, NSW, Australia; b Australian Centre for Genomic Epidemiological Microbiology, University of Technology Sydney, Ultimo, NSW, Australia

## ERRATUM

Volume 7, no. 1, e01212-21, 2022, https://doi.org/10.1128/msystems.01212-21. In Fig. 5, the color legend labeled “Source” should read, from top to bottom, “Bovine,” “Companion Animal,” “Human Other,” “Other,” “Poultry,” “Human ExPEC,” and “Porcine.” The corrected figure is shown below.

**Figure 1:**